# Development of a patient decision aid prototype on the decision to continue, reduce or discontinue antipsychotic medication following remission of first-episode psychosis

**DOI:** 10.1192/bjo.2026.11034

**Published:** 2026-05-06

**Authors:** Laurent Béchard, Olivier Corbeil, Maxime Huot-Lavoie, Olivier Roy, Sébastien Brodeur, Emmanuelle Bouchard, Joanne Martel, Natalie Pavey, Amélie M. Achim, Matthew Menear, Marc-André Roy, Sophie Lauzier, Marie-France Demers

**Affiliations:** Faculty of Nursing, Université Laval, Quebec, Canada; Faculty of Pharmacy, Université Laval, Quebec, Canada; Quebec Mental Health University Institute, CIUSSS-CN, Quebec, Canada; CERVO Brain Research Centre, Quebec, Canada; Department of Psychiatry and Neurosciences, Faculty of Medicine, Université Laval, Quebec, Canada; Department of Health Sciences, Université du Québec à Rimouski, Quebec, Canada; Independent Researcher, Quebec, Canada; VITAM Research Center in Sustainable Health, CIUSSS-CN, Quebec, Canada; Department of Family Medicine and Emergency Medicine, Faculty of Medicine, Université Laval, Quebec, Canada; CHU de Québec-Université Laval Research Centre, Quebec, Canada

**Keywords:** Decision aid, psychosis, antipsychotics, shared decision making, recovery

## Abstract

**Background:**

Shared decision-making is essential to patient-centred care, but remains underutilised in psychiatry, particularly when deciding whether to continue, reduce or stop antipsychotic medication after remission from first-episode psychosis (FEP). Existing decision aids do not fully address recovery goals such as autonomy, identity and social reintegration.

**Aims:**

To co-develop a patient decision aid (PDA) prototype that supports individuals in making the decision to continue, reduce or stop antipsychotics following remission from FEP.

**Method:**

We used a patient-centred design process informed by International Patient Decision Aid Standards (IPDAS), User Centered Design (UCD-11) and the CHIME framework. A multidisciplinary steering group – including individuals with lived experience, clinicians, and researchers – co-developed the PDA. Iterative feedback was collected from an external advisory group of patient partners, caregivers and healthcare providers (*n* = 7). Acceptability was evaluated with structured questionnaires.

**Results:**

The final prototype, structured into five sections (decision overview, personal values, risks and benefits, planning and real-life experiences), demonstrated strong acceptability across stakeholders. Ratings improved with each iteration, with version 3 receiving near-perfect scores on clarity, usefulness and balance. Users described the tool as relatable and empowering. The inclusion of real-life stories and visual decision exercises were particularly valued. However, some clinicians expressed concerns about time constraints and workflow integration.

**Conclusions:**

This recovery-oriented PDA prototype offers a practical, evidence-based resource to facilitate shared decision-making with respect to continuing, reducing or stopping antipsychotics after FEP. Although early feedback is promising, pilot testing is needed to evaluate its impact on decision quality, satisfaction and treatment outcomes.

Antipsychotic medication plays a critical role in the management of psychosis, particularly in individuals recovering from a first episode of psychosis (FEP).^1^ Standard care typically involves maintaining antipsychotic treatment for 1–5 years following remission from FEP, to reduce the risk of relapse.^2^ However, a key challenge for both individuals with FEP and clinicians arises after remission: the decision to continue or discontinue antipsychotic medication.^3,4^ This decision involves balancing the risks of relapse against the side-effects and long-term consequences of continued medication use, such as weight gain, metabolic disturbances and tardive dyskinesia.^5,6^ Moreover, how prolonged medication use affects quality of life and social functioning in the long term remains uncertain.^7^ Increasingly, psychosis management is recognised as a multidimensional process, where decisions are made not only to control symptoms, but also to support recovery goals, such as social reintegration, autonomy and the pursuit of life aspirations.^8^

In this context, shared decision-making has emerged as a key principle in modern mental healthcare.^9^ Shared decision-making is a collaborative process in which individuals and clinicians jointly explore treatment options, considering clinical evidence, individual preferences, values, and life circumstances.^10^ It is especially relevant in situations where multiple treatment options exist, each with their own risks and benefits – such as the decision to stop or continue antipsychotic medication after remission from FEP. Shared decision-making empowers patients,^11^ fosters a stronger therapeutic alliance^12^ and has been associated with improved treatment adherence and satisfaction.^13,14^ Despite these benefits, shared decision-making is underutilised in mental healthcare.^15,16^

Patient decision aids (PDAs) offer a potential solution to this challenge. PDAs are tools designed to facilitate shared decision-making by providing people with clear, accessible information about their treatment options, helping them weigh the pros and cons of each option, and clarifying their values and preferences.^17^ They have been shown to reduce decisional conflict, improve patient knowledge and enhance participation in healthcare decisions.^17^ Currently, three PDAs support decisions about antipsychotic treatment in stable non-affective psychosis.^4,18–20^ One helps individuals changing an antipsychotic medication by reviewing past experiences, prioritising symptom management, evaluating side-effects and considering administration preferences.^4^ The second PDA helps individuals with stable schizophrenia decide whether to reduce or maintain their antipsychotic treatment, specifically comparing the risks and benefits of dose reduction, monotherapy or continued polypharmacy.^19,20^ A third PDA guides decisions about continuing, adjusting or stopping antipsychotics, focusing on relapse risk, side-effects and daily functioning.^18^ Although these PDAs are valuable resources, they do not fully address the broader recovery-oriented needs of individuals in remission from FEP. Considering recovery goals – such as autonomy, social reintegration and life aspirations – is essential because these goals reflect what individuals in remission from FEP value most in their lives. Treatment decisions that align with these priorities can enhance engagement, foster a stronger sense of control and support long-term well-being.^21^ For many, decisions concerning antipsychotic medication go beyond relapse prevention; it is about reclaiming a meaningful and self-directed life.^22^

The purpose of this article is to describe the development of a PDA designed for individuals in remission from FEP to support decisions about continuing, reducing or stopping antipsychotic medication. Developed through a collaborative process, the PDA uniquely emphasises personal recovery, addressing autonomy, life goals and functional well-being alongside clinical outcomes. By focusing on the individual’s perspective, it aims to promote shared decision-making that respects both evidence-based guidance and each person’s recovery journey.

## Method

### Study design

We used a user-centred, multi-phase, mixed-methods design to develop a PDA for individuals in remission from FEP who are considering whether to continue, reduce or discontinue antipsychotic medication. The development process was guided by the 2021 International Patient Decision Aid Standards (IPDAS), including the minimum criteria for balanced, evidence-based and transparent information,^23,24^ and the CHIME recovery framework, which emphasises Connectedness, Hope, Identity, Meaning and Empowerment to ensure the PDA supports patients’ broader recovery goals.^22^ The process began with a systematic review and meta-analysis synthesising evidence on clinical outcomes associated with antipsychotic continuation or discontinuation,^7^ followed by a qualitative needs assessment to explore the decision-making process and identify key motivators and support needs.^25^ A multidisciplinary steering group and an external advisory group that included potential end users contributed throughout the development process, from conceptualisation to iterative refinement. Members of both groups were identified through purposive sampling of individuals with relevant expertise, facilitated through our professional and lived experience networks. Field testing in clinical practice has not yet been conducted, and will be the subject of future research.

### Multidisciplinary steering group

To guide the PDA’s development, we assembled a multidisciplinary steering group, comprising two clinical pharmacist researchers (L.B., M.-F.D.), one psychiatrist researcher (M.-A.R.), one researcher in psychosocial dimensions of medication use (S.L.), one mental health services researcher with expertise in shared decision-making (M.M.) and one patient partner in research (J.M.). The group met regularly throughout the development process to conceptualise the PDA, define its objectives and ensure its alignment with both clinical priorities and personal recovery goals. Throughout the meetings, the perspectives of the member with lived experience (J.M.) were actively solicited and fully integrated into all decisions. After each iterative cycle, the group reviewed updated prototypes, analysed feedback from the external advisory group, and provided input to improve clarity, relevance and usefulness. The steering group also used the IPDAS 2.0 checklist to ensure that the PDA met international standards for balance, accessibility and evidence-based content.

### External advisory group

**Fig. 1:**
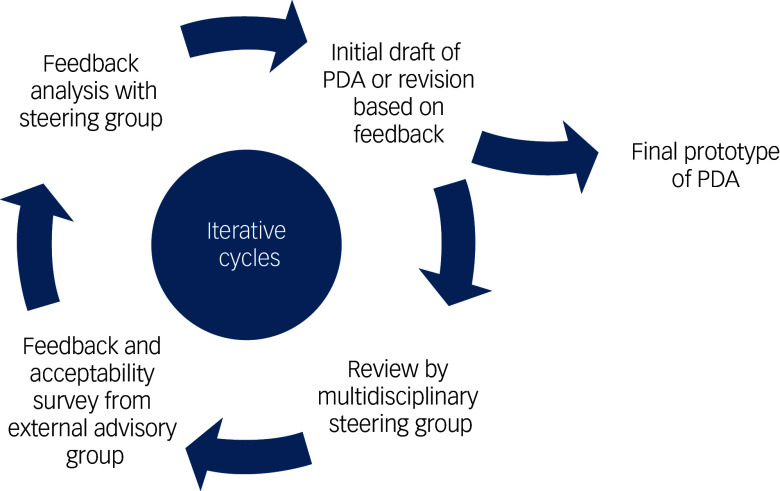
Iterative development process of the patient decision aid (PDA) prototype. The PDA was refined through repeated cycles of drafting, review by a multidisciplinary steering group and feedback from an external advisory group, until a final version was reached.

The external advisory group was composed of three patient partners in research, one family caregiver and three healthcare professionals (two psychiatrists and one nurse practitioner specialising in psychiatry: O.R., S.B., E.B.). The group contributed end-user perspectives by reviewing each version of the PDA during its development (see Fig. 1). Individually, members provided written feedback and completed structured questionnaires assessing comprehension, relevance, ease of use and perceived usefulness. Their input – grounded in lived, caregiving and clinical experience specifically related to antipsychotic decisions after remission from FEP – helped refine the content and format to better meet the needs of its intended users.

### Scope

The PDA was developed for adults in remission from non-affective or affective psychosis who are currently treated with antipsychotics, ideally after 1 year of remission from an FEP. However, it may also be used at other points in remission, with the expectation that clinicians adapt the information and discussion accordingly. The tool supports decision-making among individuals, caregivers and early intervention clinicians considering three options: discontinuing antipsychotics, continuing at full dose or reducing the dose.

### PDA content

#### Structure and frameworks

The PDA is designed for use during medical consultations, where members of the clinical team can support individuals in understanding the information provided when needed. To facilitate its use, a manual for clinicians was also developed and is available upon request. The PDA’s design and content were structured using the IPDAS minimal criteria (version 4.0), which define the minimum criteria for high-quality decision aids, including accuracy, balance, transparency and evidence-based content.^24^ The development was further informed by the User-Centered Design 11-item measure (UCD-11) checklist, which outlines best practices for user-centred design through iterative feedback, real-world relevance and involvement of potential users at multiple stages.^26^ The qualitative study helped identify key decisional needs, common motivators and the overall decision-making process, informing what content to include, how to present options and who should be involved in the discussion. The CHIME framework further grounded the tool in personal recovery principles: it shaped the value clarification section around life priorities (Hope, Meaning); guided the inclusion of examples such as returning to work or regaining autonomy; supported the use of empowering, non-directive language (Empowerment); and emphasised the importance of involving trusted individuals in the process (Connectedness).

#### Evidence selection

Development of the PDA content on treatment options and outcomes relied on robust evidence from several studies, prioritising meta-analyses or clinical trials to ensure the highest level of scientific rigour. Our content development strategy drew on prior work from our team, including a published meta-analysis on the effects of antipsychotic discontinuation in FEP,^7^ which documented key outcomes such as hospital admission rates, quality of life and recovery. Additional meta-analyses provided evidence on relapse risks, evaluated the reversibility of weight gain after antipsychotic discontinuation and assessed the risks of tardive dyskinesia and other adverse effects, thus addressing aspects of patients’ physical and mental well-being.^2,27–35^ When meta-analyses or systematic reviews were unavailable, we followed the evidence-based medicine hierarchy – prioritising randomised controlled trials, then non-randomised trials and observational studies – to select the most relevant available evidence, favouring studies conducted in FEP settings or, when necessary, among individuals with schizophrenia or antipsychotic users. The multidisciplinary steering group reviewed and discussed evidence to ensure the PDA content accurately reflected both patient and clinical priorities, with experts providing critical insights on the selected references. Relapse and hospital admission were treated as dichotomous outcomes, consistent with how these events are reported in the literature.

#### Narrative integration

To enhance relatability and support a values-based decision-making process, three narrative stories were incorporated into the PDA. These stories emphasise that there is no universally correct choice, only what is right for each individual.^25,36^ They were inspired by real-life experiences from a qualitative study participant, a member of the external advisory group and a member of the multidisciplinary steering group with lived experience. All stories were co-written with the steering group member with lived experience, and reviewed by the external advisory group to ensure authenticity, diversity and person-centredness.

#### Language and readability

The French version of the PDA was professionally translated into English by a certified translator with lived experience of psychosis (N.P.), who also reviewed the tool to ensure clarity, relevance and inclusivity. She reported that engaging with the PDA prompted personal reflection and found it meaningful, offering informal validation of its usefulness from an end-user perspective.

To ensure adequate readability, we worked with the SHeLL Editor (web-based tool; Sydney Health Literacy Lab, University of Sydney, Sydney, Australia; https://shell.techlab.works/) to identify complex terms and replace them with simpler synonyms, resulting in a more accessible version. The readability of the English versions were assessed using the readability calculator Online-Utility.org (web-based tool; https://www.online-utility.org/english/readability_test_and_improve.jsp).^37^ The grade reading level was estimated by averaging the SMOG and Gunning Fog scores, which indicate the number of years of formal education required to understand the text.

### Iterative process

The PDA was developed through a three-cycle, user-centred iterative process involving both the multidisciplinary steering group and an external advisory group (see Fig. 1). Based on initial input from the multidisciplinary steering group – focused on structure, content accuracy, clarity and alignment with recovery-oriented and shared decision-making principles – version 1 of the PDA prototype was created. This version was then sent individually to each member of the external advisory group for review. They provided written feedback and completed structured questionnaires. All feedback was systematically documented in a tracking table (see Supplementary Material 1), where each comment was linked to either a corresponding modification in the next version or a justification when no change was made. All changes made in response to advisory group feedback were explicitly highlighted in the updated prototype to ensure transparency. The revised version was then returned to the steering group, discussed and refined in the next iteration. This cycle of feedback, documentation and revision was repeated three times until consensus was reached on the final version of the PDA.

### Acceptability assessment

To evaluate acceptability, we administered two distinct questionnaires adapted from the Ottawa Hospital Research Institute^38^ to members of the external advisory group. Members with lived experience (*n* = 4) rated each section of the PDA (such as ‘What is a patient decision aid?’, ‘What are the pros and cons of each option?’, ‘How does this choice align with your priorities?’ and ‘Create an action plan’), using a five-point scale ranging from ‘poor’ to ‘excellent’. Members with clinical expertise (*n* = 3), on the other hand, completed a 14-item satisfaction questionnaire using a five-point Likert scale (1 = ‘strongly disagree’ to 5 = ‘strongly agree’) to assess the tool’s usability and perceived effectiveness (see Supplementary Material 2–5). Responses were summarised using descriptive statistics.

Additionally, an informal meeting was held with 31 clinicians from the Clinique Notre-Dame des Victoires, an early intervention clinic for FEP in Quebec City that follows approximately 250 individuals, to present the final version of the PDA and gather further feedback. Attendees included case managers from various professional backgrounds (such as nursing, occupational therapy and social work), one peer support worker, pharmacists, psychiatrists and psychologists.

### Ethics statement

The authors assert that all procedures contributing to this work comply with the ethical standards of the relevant national and institutional committees on human experimentation and with the Helsinki Declaration of 1975, as revised in 2013. All procedures involving human subjects/patients were approved by the CÉR-S en neurosciences et santé mentale du CIUSSS-CN (approval number #MP-13-2021-2325). Formal consent was not required for this study, as determined by the institutional research ethics board. However, all participants involved in the steering group and external advisory group still provided written agreement to participate via email.

## Results

### IPDAS criteria and user-centredness

The prototype developed (see Supplementary Material) meets all IPDAS version 4.0 qualifying and certification criteria,^24^ and 21 out of 28 of the quality criteria. Similarly, 9 of the 11 UCD-11 items were met.^23,26^ The remaining criteria were not achieved solely because the prototype has not yet undergone field testing with patients and clinicians (see Supplementary Material 6 and 7).

### Feedback and iterative revisions

Across three multidisciplinary steering group meetings, rigorous notes were taken and all suggestions were carefully reviewed and integrated. This iterative process led to substantial improvements in the PDA’s content, tone and format. Revisions focused on enhancing clarity, reducing stigma, improving personalisation and supporting patient engagement at different stages of the decision-making process. Drawing on themes from our qualitative study currently under review, discussions also explored how self-stigma – and related experiences, such as internalised negative views of psychosis, medication as a reminder of illness or a perceived shift in identity – might influence the desire to stop medication. Additional materials, such as a clinician guide, were also developed to support implementation.

Across the three versions of the PDA, a total of 143 comments were submitted by the external advisory group. For version 1, 101 comments were received, with 66 (65%) leading to revisions aimed at improving clarity, refining terminology (e.g. remission, treatment options) and better aligning examples with recovery goals. Suggestions not incorporated were typically beyond the tool’s scope, redundant or inconsistent with its purpose. In version 2, 19 comments were received, of which 14 (74%) prompted changes, including the addition of experiential knowledge, clarification of clinical concepts (e.g. insight, defined daily dose) and population details. Broader issues, such as comorbidities and layout, were noted, but not fully addressed. For version 3, 23 comments were submitted, resulting in 12 (52%) modifications focused on wording and clarifications, whereas broader suggestions like expanding to other diagnoses or including external guides were not adopted because of scope limitations.

The final version of the PDA was presented to 31 clinicians from early intervention services during an informal meeting. Although no formal evaluation was conducted and no specific comments were received, the overall reception was broadly positive, reinforcing the tool’s perceived relevance and usability in clinical practice.

### Description of the final PDA

The final prototype is a 15-page, paper-based tool designed to be introduced during clinical encounters. It includes a definition of patient decision aids, rationale for the decision, presentation of the three main options (continue, reduce or stop antipsychotics), pros and cons by domain (mental health, life impact, short- and long-term side-effects), values clarification, summary alignment with user priorities, action planning and three real-life narratives (see Supplementary Material). Although initially presented in a clinical setting, the tool is designed to support ongoing reflection afterward. It is recovery-oriented in that it helps users explore how each option aligns with their life goals, encourages hope through concrete examples (e.g. returning to work, regaining autonomy) and invites the involvement of trusted individuals in the decision-making process, among other features designed to support personal recovery.^22^

### Acceptability

The PDA received progressively higher ratings across iterations, with the final version scoring highest on nearly all acceptability items. Among respondents with clinical expertise, the largest improvements (0.7 to 1.1 points) were observed for items related to ease of use, clarity, perceived reliability, adaptability, alignment with clinical practice, anticipated benefit/harm ratio and support for informed decision-making (see Table 1). Other items showed minimal change (0 to 0.3 points), but were already rated positively, with average scores around 4, reflecting agreement on compatibility with usual practice, suitability for value-laden choices and partial use of the tool. In contrast, two items – time efficiency (mean: 3.3) and avoiding changes to clinical routines (mean: 3.7) – consistently received lower scores. These findings suggest that although the PDA was viewed as useful and relevant, clinicians did not anticipate time savings and perceived that its use might require adapting existing workflows.

**Table 1:**
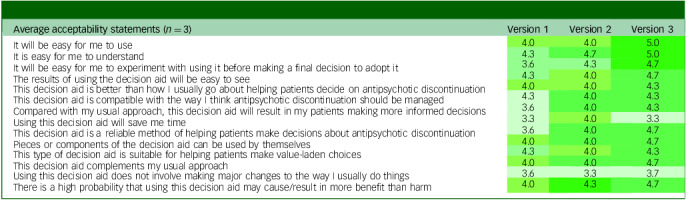
Average acceptability ratings from healthcare providers in the external advisory group across iterative versions

Each value represents the mean score per statement for a given version (1 = strongly disagree; 5 = strongly agree).

According to the acceptability questionnaire completed by participants with lived experience (see Table 2), the greatest improvements (1.5 to 2.5 points) were observed for sections on the pros and cons of each option, values clarification, action planning and real-life experiences. The narrative stories section showed the largest gain, increasing from a mean score of 2.5 to 5.0 after the addition of a third story and revisions to better reflect diverse experiences. Moderate improvements (0.7 to 1.0 points) were noted for sections such as ‘What is a patient decision aid?’, ‘Why is this decision important?’ and ‘How will this decision be made?’, all of which reached perfect scores in the final version.

**Table 2:**
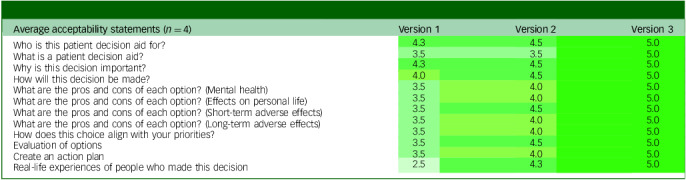
Average acceptability ratings from members with lived experience in the external advisory group across iterative versions

Each value represents the mean score per section of the patient decision aid for a given version (1 = mediocre; 5 = excellent).

The final version of the PDA was also considered balanced, with most participants finding the length and amount of information appropriate (Table 3). Six out of seven respondents stated they would have found the tool very or extremely useful when facing the decision to continue or discontinue antipsychotics. Supporting its accessibility, the plain-language version of the PDA achieved an average readability level equivalent to grade 9 (Gunning Fog = 9.00; SMOG = 9.40).

**Table 3:** Perceived acceptability of the final version of the decision aid among external advisory group members

Acceptability items	*n* (%)
The length of the decision aid was just right	6 (86)
The amount of information was just right	6 (86)
I found the decision aid balanced	7 (100)
I would have found this decision aid ‘very or extremely useful’ when making the decision to discontinue or continue antipsychotics	6 (86)

Proportions reflect the number and percentage of members (*n* = 7) who agreed with each acceptability item regarding the final version of the patient decision aid.

The external advisory group described the prototype as a useful, reassuring and accessible resource to support decision-making (see Supplementary Material 8). They highlighted its ability to foster open and collaborative dialogue among patients, families and clinicians. The following quotes are free translations from French: a caregiver shared, ‘This tool would have helped me better support my sons when this issue became significant for them’. A healthcare professional noted, ‘It makes things easier … it provides the necessary information to make an informed choice’. Users also appreciated the figures and tables and the way the tool encourages reflection on values: ‘It facilitates decision-making through its visual format and the decision balance exercise, which is well-supported by evidence and peer testimonies’. Another participant commented, ‘I would probably have felt more secure in my decision receiving this document, because being encouraged to make an informed choice shows that my opinion and point of view matter’.

## Discussion

This article presents the systematic development of a novel, evidence-based PDA to support individuals in remission from an FEP as they consider whether to continue, reduce or discontinue antipsychotic medication. In addition to addressing relapse prevention, the tool emphasises personal recovery goals such as autonomy, hope and social reintegration – priorities frequently reported by individuals with lived experience.^38^ Its development followed a rigorous, iterative and user-centred process, aligned with IPDAS standards and guided by input from a multidisciplinary team and stakeholders with both clinical and lived experience.

### Key contributions and innovations

Although several PDAs exist to support decisions about antipsychotic treatment,^4,18–20^ ours is the first developed specifically for individuals in remission from an affective or non-affective FEP. Unlike tools focused on switching or reducing an antipsychotic medication, or designed for broader psychosis populations, our PDA addresses the full range of options (continuing, reducing or discontinuing) within the context of early recovery. This is particularly important, as some individuals express a desire to stop treatment – a need previously acknowledged, but not directly addressed in existing tools.^4^ In our PDA, discontinuation is stated explicitly because many individuals are unaware that it is even a possibility, whereas in practice, this option is operationalised through a gradual taper.

Our PDA places stronger emphasis on personal recovery goals, involves trusted others in the decision-making process, and encourages ongoing reflection beyond the clinical encounter. When disagreement occurs, the PDA helps make differences in preferences and concerns explicit so that the person and clinician can explore them together and work toward a mutually acceptable plan. It was co-developed with individuals with lived experience and guided by the CHIME framework, ensuring that recovery-oriented values are embedded throughout. Notably, our PDA is the only one to include narrative stories – developed in response to needs identified in our qualitative study – which help users relate to different recovery paths and support values-based deliberation.^25^

Although our tool shares aims with the Antipsychotic Medication Decision Aid (APM-DA)^18,38–40^ – the only other PDA specifically addressing continuation, adjustment or discontinuation post-remission – there are key differences. The APM-DA was pilot-tested in an FEP setting with good results,^38^ but it was developed with a broader psychosis population. In contrast, our PDA was designed from the ground up for FEP, a population with typically better outcomes and for whom the evidence base is more limited.^40^ This makes the clear communication of uncertainty especially important, and highlights the need for tailored decision support in early intervention settings.

### Strengths and limitations

This study has several strengths. The PDA was developed using a transparent, iterative and user-centred process grounded in established frameworks (UCD-11, IPDAS and CHIME), with meaningful input from individuals with lived experience, clinicians and researchers. It is one of the few tools specifically designed for individuals in remission from an FEP, integrating both clinical evidence and recovery-oriented perspectives. The PDA supports reflection beyond the clinical encounter and addresses a wide range of outcomes, from relapse prevention to personal goals such as autonomy and social reintegration. Additionally, the inclusion of narrative stories based on real experiences responds to needs identified in our qualitative research, and enhances the tool’s relatability and potential to reduce stigma. Finally, the development process is documented in detail, with successive versions and justifications for changes available in the Supplementary Material.

This work has some limitations. Although the PDA benefited from strong participation by individuals with lived experience, the composition of the advisory and steering groups lacked ethnocultural diversity, as all members were Quebec-born. This may have limited the range of perspectives integrated into the tool. Future development should aim to include more diverse voices to enhance cultural sensitivity and inclusivity. In addition, although the narrative stories were revised to improve relatability, they may not reflect the full spectrum of patient experiences. Some clinicians expressed theoretical concerns about time demands and workflow integration based solely on reviewing the prototype, highlighting the need for future implementation research once the PDA is field-tested. Finally, although the tool was developed for individuals in remission from FEP, its applicability to other diagnostic groups remains to be tested.

In conclusion, this recovery-oriented PDA provides an evidence-based tool to help individuals in remission from FEP make informed decisions about the continuation or discontinuation of antipsychotic treatment. Rather than promoting a single ‘right’ choice, it encourages reflection on personal values and goals, with clinicians providing support through empathy, clear communication and the best available evidence. Next steps include pilot-testing the PDA in early intervention settings through a randomised trial to evaluate its impact on decision quality, patient satisfaction, treatment engagement, clinical outcomes such as rates of relapse, and recovery outcomes. Future adaptations may include tailoring the tool for individuals with chronic psychosis and developing digital formats to improve accessibility.

## Supporting information

10.1192/bjo.2026.11034.sm001 Béchard et al. supplementary material 1 Béchard et al. supplementary material

10.1192/bjo.2026.11034.sm002 Béchard et al. supplementary material 2 Béchard et al. supplementary material

10.1192/bjo.2026.11034.sm003 Béchard et al. supplementary material 3 Béchard et al. supplementary material

## Data Availability

The data that support the findings of this study are available from the corresponding author, L.B., upon reasonable request.

## Supplementary material

The supplementary material is available online at https://doi.org/10.1192/bjo.2026.11034. The supplementary materials for this paper are published under a CC-BY NC licence: © The Author(s), 2026. Published by Cambridge University Press on behalf of Royal College of Psychiatrists. This is an Open Access article, distributed under the terms of the Creative Commons Attribution-NonCommercial licence (https://creativecommons.org/licenses/by-nc/4.0/), which permits non-commercial re-use, distribution, and reproduction in any medium, provided the original work is properly cited. The written permission of Cambridge University Press must be obtained for commercial re-use.

## References

[1] Keepers GA , Fochtmann LJ , Anzia JM , Benjamin S , Lyness JM , Mojtabai R , et al. The American Psychiatric Association practice guideline for the treatment of patients with schizophrenia. Am J Psychiatry 2020; 177: 868–72. 32867516 10.1176/appi.ajp.2020.177901

[2] Hui CLM , Chen EYH , Verma S , Tagata H , Mizuno M , Liu CC , et al. Guidelines for discontinuation of antipsychotics in patients who recover from first-episode schizophrenia spectrum disorders: derived from the aggregated opinions of Asian network of early psychosis experts and literature review. Int J Neuropsychopharmacol 2022; 25: 737–58. 35451023 10.1093/ijnp/pyac002 PMC9515132

[3] Thomas EC , Suarez J , Lucksted A , Siminoff L , Hurford I , Dixon L , et al. Treatment decision-making needs among emerging adults with early psychosis. Early Interv Psychiatry 2022; 16: 78–90. 33599089 10.1111/eip.13134 PMC9116145

[4] Kaar SJ , Gobjila C , Butler E , Henderson C , Howes OD. Making decisions about antipsychotics: a qualitative study of patient experience and the development of a decision aid. BMC Psychiatry 2019; 19: 309. 31646985 10.1186/s12888-019-2304-3 PMC6806500

[5] Hui CLM , Lam BST , Lee EHM , Chan SKW , Chang WC , Suen YN , et al. Perspective on medication decisions following remission from first-episode psychosis. Schizophr Res 2020; 225: 82–9. 32115314 10.1016/j.schres.2020.02.007

[6] Béchard L , Corbeil O , Malenfant E , Lehoux C , Stip E , Roy MA , et al. Une approche de la psychopharmacologie des premiers épisodes psychotiques axée sur le rétablissement. [Psychopharmacology of first episode psychosis: an approach based on recovery.] Sante Ment Que 2021; 46: 113–37. 35617496

[7] Béchard L , Desmeules C , Bachand L , Huot-Lavoie M , Corbeil O , Anderson E , et al. The effects of antipsychotic discontinuation or maintenance on the process of recovery in remitted first-episode psychosis patients - a systematic review and meta-analysis of randomized controlled trials. Eur Psychiatry 2024; 67: e13. 38250810 10.1192/j.eurpsy.2024.5 PMC10897830

[8] Warner R. Recovery from schizophrenia and the recovery model. Curr Opin Psychiatry 2009; 22: 374–80. 19417668 10.1097/YCO.0b013e32832c920b

[9] Slade M. Implementing shared decision making in routine mental health care. World Psychiatry 2017; 16: 146–53. 28498575 10.1002/wps.20412 PMC5428178

[10] Coulter A , Collins A. Making Shared Decision-Making a Reality. No Decision about Me, without Me. The King’s Fund, 2011 (https://assets.kingsfund.org.uk/f/256914/x/73b4098901/making_shared_decisions_making_reality_july_2011.pdf).

[11] Stovell D , Morrison AP , Panayiotou M , Hutton P. Shared treatment decision-making and empowerment-related outcomes in psychosis: systematic review and meta-analysis. Br J Psychiatry 2016; 209: 23–8. 27198483 10.1192/bjp.bp.114.158931

[12] Matthias MS , Fukui S , Kukla M , Eliacin J , Bonfils KA , Firmin RL , et al. Consumer and relationship factors associated with shared decision making in mental health consultations. Psychiatr Serv 2014; 65: 1488–91. 25220249 10.1176/appi.ps.201300563 PMC12171972

[13] Renes JW , Metz MJ , Nolen WA , Hoogendoorn AW , Kupka RW , Regeer EJ. Shared decision-making in the treatment of bipolar disorder: findings from a nationwide naturalistic cohort study in everyday clinical practice. Soc Psychiatry Psychiatr Epidemiol 2025; 60: 1489–97. 39377952 10.1007/s00127-024-02761-8

[14] Pérez-Revuelta JI , González-Sáiz F , Pascual-Paño JM , Mongil-San Juan JM , Rodríguez-Gómez C , Muñoz-Manchado LI , et al. Shared decision making with schizophrenic patients: a randomized controlled clinical trial with booster sessions (DECIDE Study). Patient Educ Couns 2023; 110: 107656. 36807126 10.1016/j.pec.2023.107656

[15] Hamann J , Mendel R , Cohen R , Heres S , Ziegler M , Bühner M , et al. Psychiatrists use of shared decision making in the treatment of schizophrenia: patient characteristics and decision topics. Psychiatr Serv 2009; 60: 1107–12. 19648199 10.1176/ps.2009.60.8.1107

[16] Coulter A. Shared decision making: everyone wants it, so why isn’t it happening? World Psychiatry 2017; 16: 117–8. 28498596 10.1002/wps.20407 PMC5428189

[17] Stacey D , Légaré F , Lewis K , Barry MJ , Bennett CL , Eden KB , et al. Decision aids for people facing health treatment or screening decisions. Cochrane Database Syst Rev 2017; 4: Cd001431. 28402085 10.1002/14651858.CD001431.pub5 PMC6478132

[18] Zisman-Ilani Y , Shern D , Deegan P , Kreyenbuhl J , Dixon L , Drake R , et al. Continue, adjust, or stop antipsychotic medication: developing and user testing an encounter decision aid for people with first-episode and long-term psychosis. BMC Psychiatry 2018; 18: 142. 29788933 10.1186/s12888-018-1707-x PMC5963160

[19] Aoki Y , Takaesu Y , Matsui K , Tokumasu T , Tani H , Takekita Y , et al. Development and acceptability testing of a decision aid for considering whether to reduce antipsychotics in individuals with stable schizophrenia. Neuropsychopharmacol Rep 2023; 43: 391–402. 10.1002/npr2.12366 PMC10496039 37452456

[20] Dixon LB , Holoshitz Y , Nossel I. Treatment engagement of individuals experiencing mental illness: review and update. World Psychiatry 2016; 15: 13–20. 26833597 10.1002/wps.20306 PMC4780300

[21] Leamy M , Bird V , Le Boutillier C , Williams J , Slade M. Conceptual framework for personal recovery in mental health: systematic review and narrative synthesis. Br J Psychiatry 2011; 199: 445–52. 22130746 10.1192/bjp.bp.110.083733

[22] Witteman HO , Maki KG , Vaisson G , Finderup J , Lewis KB , Dahl Steffensen K , et al. Systematic development of patient decision aids: an update from the IPDAS collaboration. Med Decis Making 2021; 41: 736–54. 34148384 10.1177/0272989X211014163 PMC8664088

[23] Joseph-Williams N , Newcombe R , Politi M , Durand MA , Sivell S , Stacey D , et al. Toward minimum standards for certifying patient decision aids: a modified delphi consensus process. Med Decis Making 2014; 34: 699–710. 23963501 10.1177/0272989X13501721

[24] Béchard L , Anderson E , Corbeil O , Huot-Lavoie M , Brodeur S , Massé C , et al. Factors shaping the decision-making process to continue or discontinue antipsychotics: a qualitative study of individuals in remission from first-episode psychosis. BJPsych Open 2024; 11: e193. 10.1192/bjo.2025.10817 PMC12451548 40904211

[25] Witteman HO , Vaisson G , Provencher T , Chipenda Dansokho S , Colquhoun H , Dugas M , et al. An 11-item measure of user- and human-centered design for personal health tools (UCD-11): development and validation. J Med Internet Res 2021; 23: e15032. 33724194 10.2196/15032 PMC8074832

[26] Speyer H , Westergaard C , Albert N , Karlsen M , Stürup AE , Nordentoft M , et al. Reversibility of antipsychotic-induced weight gain: a systematic review and meta-analysis. Front Endocrinol (Lausanne) 2021; 12: 577919. 34393989 10.3389/fendo.2021.577919 PMC8355990

[27] Kishi T , Ikuta T , Matsui Y , Inada K , Matsuda Y , Mishima K , et al. Effect of discontinuation v. maintenance of antipsychotic medication on relapse rates in patients with remitted/stable first-episode psychosis: a meta-analysis. Psychol Med 2019; 49: 772–9. 29909790 10.1017/S0033291718001393

[28] Leucht S , Tardy M , Komossa K , Heres S , Kissling W , Salanti G , et al. Antipsychotic drugs versus placebo for relapse prevention in schizophrenia: a systematic review and meta-analysis. Lancet 2012; 379: 2063–71. 22560607 10.1016/S0140-6736(12)60239-6

[29] Carbon M , Kane JM , Leucht S , Correll CU. Tardive dyskinesia risk with first- and second-generation antipsychotics in comparative randomized controlled trials: a meta-analysis. World Psychiatry 2018; 17: 330–40. 30192088 10.1002/wps.20579 PMC6127753

[30] Ceraso A , Lin JJ , Schneider-Thoma J , Siafis S , Heres S , Kissling W , et al. Maintenance treatment with antipsychotic drugs in schizophrenia: a cochrane systematic review and meta-analysis. Schizophr Bull 2022; 48: 738–40. 35556140 10.1093/schbul/sbac041 PMC9212092

[31] Thompson A , Winsper C , Marwaha S , Haynes J , Alvarez-Jimenez M , Hetrick S , et al. Maintenance antipsychotic treatment versus discontinuation strategies following remission from first episode psychosis: systematic review. BJPsych Open 2018; 4: 215–25. 29988997 10.1192/bjo.2018.17 PMC6034451

[32] Brandt L , Schneider-Thoma J , Siafis S , Efthimiou O , Bermpohl F , Loncar L , et al. Adverse events after antipsychotic discontinuation: an individual participant data meta-analysis. Lancet Psychiatry 2022; 9: 232–42. 35183280 10.1016/S2215-0366(22)00014-1

[33] Rodolico A , Siafis S , Bighelli I , Samara MT , Hansen WP , Salomone S , et al. Antipsychotic dose reduction compared to dose continuation for people with schizophrenia. Cochrane Database Syst Rev 2022; 11: Cd014384. 36420692 10.1002/14651858.CD014384.pub2 PMC9685497

[34] Schmidt HM , Hagen M , Kriston L , Soares-Weiser K , Maayan N , Berner MM. Management of sexual dysfunction due to antipsychotic drug therapy. Cochrane Database Syst Rev 2012; 11: Cd003546. 23152218 10.1002/14651858.CD003546.pub3 PMC7003677

[35] Shaffer VA , Brodney S , Gavaruzzi T , Zisman-Ilani Y , Munro S , Smith SK , et al. Do personal stories make patient decision aids more effective? An update from the international patient decision aids standards. Med Decis Making 2021; 41: 897–906. 34027739 10.1177/0272989X211011100

[36] Online-Utility.org. *Readability Calculator*. Mladen Adamovic, 2020 (https://www.online-utility.org/english/readability_test_and_improve.jsp).

[37] O’Connor AM , Cranney A. *User Manual – Acceptability*. Ottawa Hospital Research Institute, 2002 (https://decisionaid.ohri.ca/docs/develop/User_Manuals/UM_Acceptability.pdf).

[38] Zisman-Ilani Y , Parker M , Thomas EC , Suarez J , Hurford I , Bowen A , et al. Usability and feasibility of the antipsychotic medication decision aid in a community program for first-episode psychosis. Psychiatr Serv 2024; 75: 807–11. 10.1176/appi.ps.20230230 38477836

[39] Maas IL , Bohlken MM , Gangadin SS , Rosema BS , Veling W , Boonstra N , et al. Personal recovery in first-episode psychosis: Beyond clinical and functional recovery. Schizophr Res 2024; 266: 32–40. 38367610 10.1016/j.schres.2024.02.005

[40] Simonsen C , Åsbø G , Slade M , Wold KF , Widing L , Flaaten CB , et al. A good life with psychosis: rate of positive outcomes in first-episode psychosis at 10-year follow-up. Psychol Med 2024; 54: 2112–21. 38389456 10.1017/S0033291724000205 PMC11413337

